# 共价有机框架材料在毛细管电色谱中的应用进展

**DOI:** 10.3724/SP.J.1123.2023.04005

**Published:** 2023-10-08

**Authors:** Guoxiu WANG, Yonglei CHEN, Wenjuan LÜ, Hongli CHEN, Xingguo CHEN

**Affiliations:** 1.兰州大学化学化工学院, 甘肃 兰州 730000; 1. College of Chemistry and Chemical Engineering, Lanzhou University, Lanzhou 730000, China; 2.北京市产品质量监督检验研究院, 北京 101300; 2. Beijing Products Quality Supervision and Inspection Institute, Beijing 101300, China

**Keywords:** 共价有机框架, 毛细管电色谱, 分离, 固定相, 综述, covalent organic frameworks, capillary electrochromatography (CEC), separation, stationary phases, review

## Abstract

毛细管电色谱(CEC)因兼具高效液相色谱(HPLC)的高选择性和毛细管电泳(CE)的高分离效率而受到越来越多研究者的关注。在毛细管电色谱中,选择合适的固定相材料对获得优异的分离效果起着十分重要的作用。近年来,多种新型材料如氧化石墨烯、蛋白质、金属有机框架(MOFs)及共价有机框架(COFs)等被作为固定相应用于毛细管电色谱领域以期获得更好的分离性能,同时拓展毛细管电色谱的应用范围。其中,COFs因具有孔隙率高、比表面积大、高稳定性、孔径可调和可设计性强等独特性质,在毛细管电色谱领域显示出了巨大的应用前景。鉴于此,本文对2016-2023年间COFs在毛细管电色谱领域的研究进展进行了综述,包括COFs毛细管电色谱柱的分类和制备方法,以及基于COFs固定相的毛细管电色谱技术在环境内分泌干扰物、农药、芳香族化合物、氨基酸及药物分离领域中的应用及分离机理等内容。最后对发展基于COFs固定相的毛细管电色谱应努力解决的问题和该技术未来的发展方向进行了分析和展望。

## Abstract (en)

Capillary electrochromatography (CEC) has received increased attention from the academic community because it combines the excellent selectivity of high performance liquid chromatography (HPLC) and the high efficiency of capillary electrophoresis (CE). Selecting the most appropriate stationary phase material is crucial to achieve better separation effects in CEC. In recent years, a considerable number of materials, such as graphene oxide, proteins, metal organic frameworks, and covalent organic frameworks (COFs), have been widely used as stationary phases in CEC to further improve its separation performance and extend its scope of potential applications. Among these materials, COFs have shown great application prospects in CEC owing to their unique properties, which include high porosity, large surface area, excellent stability, tunable pore size, and high designability of the framework structure. This review systematically summarizes published papers on the development and application of COFs in CEC from 2016 to 2023. First, two COF-based capillary columns (i. e., open-tube CEC columns and monolithic CEC columns) and their preparation methods are introduced. Second, the applications of CEC based on COF stationary phases in the separation of environmental endocrine disruptors, pesticides, aromatic compounds, amino acids, and drugs, particularly chiral drugs, are systematically summarized. The separation mechanism of CEC based on COF stationary phases is also introduced. At present, the good separation ability of COF-based CEC is mainly attributed to two factors: 1) The size exclusion effect of the pores of the COF stationary phase. Because of differences in the sizes of their organic molecular building units and side chains, COFs have varying pore sizes and topological structures. Thus, target analytes smaller than the pores of the COFs can enter the frameworks and interact with them during separation. On the other hand, target analytes larger than the pores of the COFs cannot enter the frameworks and interact with them during separation; thus, they can be separated. 2) The interactions between the target analytes and side chains (e. g., hydrophobic interactions, hydrogen bonding, *π-π* interactions, etc.) of the COFs. Since COFs usually contain alkyl side chains, aromatic structures, and oxygen and/or nitrogen atoms with high electronegativity, various interactions could occur between the COFs and target analytes. Finally, directions for the future development and strategic application of CEC based on COF stationary phases are proposed. We believe that future research in CEC based on COF stationary phases should focus on the following aspects: 1) The use of cheminformatics to design and construct COFs to improve the efficiency of COF capillary column preparation; 2) the development of milder methods to synthesize COFs that can meet the requirements of high performance COF capillary columns; and 3) in-depth research to explore the separation mechanism of CEC based on COF stationary phases to provide theoretical guidance for developing CEC methods suitable for the separation and analysis of complex samples.

毛细管电色谱是一种以负载固定相(通过填充、涂覆、键合或交联法制备)的毛细管为分离通道,利用电渗流驱动流动相,在高电场强度下进行分离分析的技术^[1]^。毛细管电色谱结合了毛细管电泳及高效液相色谱的优势,具有分离效率高、选择性好、分析时间短、样品和流动相消耗低等特点,受到了科研工作者的广泛关注。迄今为止,毛细管电色谱已被广泛应用于药物分析^[2⇓-4]^、环境分析^[5]^及食品安全^[6]^等领域。近年来,随着材料科学的迅猛发展及实际工作的需要,多种新型材料如金属有机框架(MOFs)^[7⇓-9]^、共价有机框架(COFs)^[10]^、多孔有机聚合物(POPs)^[11]^及纳米颗粒^[12,13]^等,在毛细管电色谱领域表现出了巨大的应用前景。

COFs是由有机分子构建基元通过共价键连接而成的晶体有机多孔材料,具有化学及热稳定性高、孔径可调、易于功能化等独特性质^[14⇓⇓-17]^,这些优良的物理化学特性赋予了COFs在毛细管电色谱领域巨大的应用潜力,已经成为一种理想的新型固定相材料^[18⇓-20]^。本文简要综述了2016-2023年间COFs在毛细管电色谱中的研究进展并对该领域今后的发展进行了展望。

## 1 COFs毛细管电色谱柱的制备

COFs毛细管电色谱柱的制备是COFs作为固定相用于毛细管电色谱分离的基础。目前,COFs毛细管电色谱柱分为开管毛细管柱及整体毛细管柱,其制备方法主要分为后修饰法及原位合成法。

### 1.1 开管毛细管电色谱柱的制备

#### 1.1.1 后修饰制备法

后修饰制备法是指将COFs悬浊液引入预修饰的毛细管,通过COFs上的残留基团与硅烷偶联剂末端基团之间的化学反应形成化学键或通过物理吸附作用,将COFs固定于毛细管内壁^[21,22]^。另外,也可以使用硅烷偶联剂首先对COFs进行修饰,然后利用硅烷偶联剂中的硅烷基与毛细管内壁的硅羟基之间的反应将COFs固定于毛细管内壁^[23,24]^。一般所使用的硅烷偶联剂末端基团为氨基及环氧基。此种方法的优点在于可以方便地制备高结晶度的COFs固定相。需指出的是,由于COFs的溶解性一般较差^[25⇓-27]^,将其悬浊液通入毛细管时容易引起毛细管堵塞,因此需要对COFs悬浊液进行超声处理,使其尽可能地分散于溶液中。

2016年,本课题组^[22]^将COF-LZU1甲苯溶液引入经3-缩水甘油醚氧基丙基三甲氧基硅烷(GLYMO)修饰的毛细管中,利用COF-LZU1中残留的氨基与偶联剂末端环氧基之间的化学反应制备了COF-LZU1开管毛细管柱,其制备过程如图1所示。2018年,Ye课题组^[23]^用对苯二甲醛和三聚氰胺通过溶剂热法制备了COF SNW-1(Schiff base network-1),并利用3-氨基丙基三乙氧基硅烷(APTES)对其进行改性后引入预处理的毛细管中,制备了SNW-1开管毛细管柱。2021年,Ye课题组^[28]^利用三醛基间苯三酚(Tp)和四(氨基苯基)甲烷(TAM)借助超声辅助法合成了COF TpTAM并通过硅烷偶联剂APTES对其改性,然后引入预处理的毛细管中制备了COF TpTAM开管毛细管柱。Yan课题组^[29]^利用*β*-环糊精COF(*β*-CD COF)上的氨基与涂敷在毛细管内壁上的聚多巴胺表面基团之间的Michael加成反应,将*β*-CD COF涂敷于毛细管内壁,制备了*β*-CD COF毛细管。2022年,本课题组^[30]^利用2,5-二甲氧基苯-1,4-二甲醛(DA)和(*S*)-1,3,5-三(4-氨基苯基)-2-(2甲基丁氧基)苯(TD)的缩合反应制备了一种新型手性DA-TD COF并将其引入到硅烷偶联剂GLYMO预修饰的毛细管中,制得了手性DA-TD COF开管毛细管柱。2022年,Ji课题组^[21]^分别利用1,4-丁磺酸内酯及(+)-二乙酰基-L-酒石酸酐对合成的*β*-CD COF_BPDA_进行修饰,然后将其引入经3-(甲基丙烯酰氧)丙基三甲氧基硅烷修饰的毛细管中制得了两种功能化*β*-CD COF_BPDA_开管毛细管柱。

**图 1:**
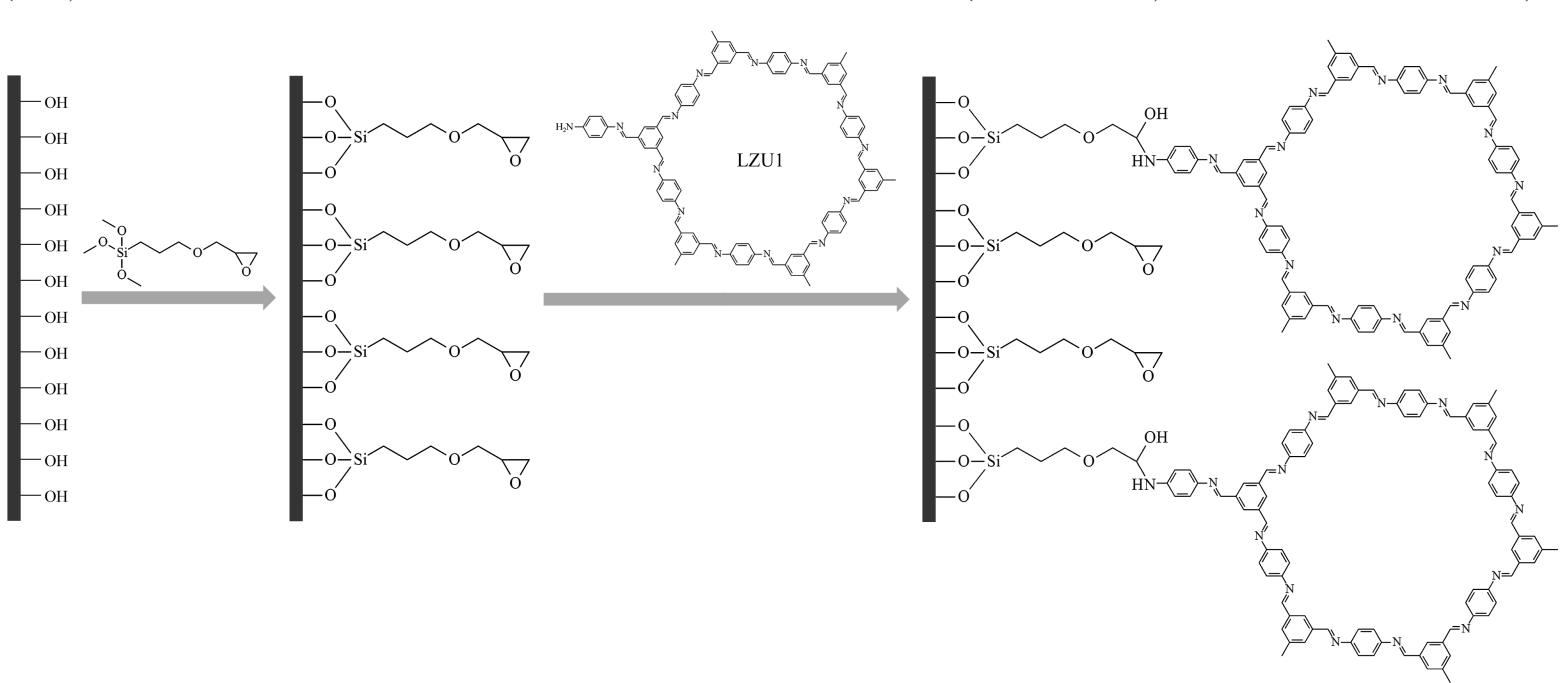
COF-LZU1涂层毛细管柱制备示意图^[22]^

#### 1.1.2 原位制备法

原位制备法是指将有机分子构建基元溶液引入到预处理后的毛细管内,在一定条件下直接在毛细管内合成COFs,并通过其有机分子构建基元与预修饰于毛细管内壁上的硅烷偶联剂之间的化学反应形成化学键或通过物理吸附作用,将COFs固定于毛细管内壁的制备开管毛细管柱的方法^[31⇓⇓⇓-35]^。与后修饰制备法相同,所使用位于毛细管内壁上的硅烷偶联剂末端基团一般为氨基及环氧基。相比于后修饰制备法,原位制备法引入有机分子构建基元溶液既可以避免毛细管堵塞,又可以使COFs均匀固定于毛细管内壁;但此方法仅适用于合成条件相对温和的COFs涂层毛细管的制备。

2016年,Chen课题组^[31]^利用向毛细管内逐步引入多巴胺溶液及有机分子构建基元2,3,6,7,10,11-六羟基三苯(HHTP)和对苯二硼酸(BDBA)的混合溶液,通过在聚多巴胺层上逐层原位生长含硼的COF-5涂层的方式,原位制备了多层COF-5开管毛细管柱。2020年,本课题组^[32]^将含有有机分子构建基元四(4-氨基苯基)甲烷及对苯二甲醛的1,4-二氧六环溶液引入至3-氨基丙基三甲氧基硅烷(APTMS)改性后的毛细管中,在毛细管内壁原位制备了三维COF-300涂层,其制备过程如图2所示。2021年,Chen课题组^[33]^利用原位生长法通过1,3,5-三(4-氨苯基)苯(TAPB)和2,5-二乙烯基-1,4-苯二甲醛(DVA)制备了COF-V开管毛细管柱。2022年,Zhang课题组^[34]^将有机分子构建基元三醛基间苯三酚、4,4'-二氨基联苯(BD)及离子液1-氨丙基-3-甲基咪唑溴盐(APMim^+^Br^-^)同时引入到APTES氨基功能化毛细管中,利用原位制备法制备了ILs@COFs开管毛细管柱。同年,本课题组^[35]^用四(4-甲酰基苯)甲烷(TFPM)和对苯二胺(PDA)通过溶剂热法实现了球形三维COFs 3D-IL-COFs的合成,在此基础上利用原位生长法制备了涂层均匀、重现性好、稳定性高的三维3D-IL-COFs开管毛细管。

**图 2:**
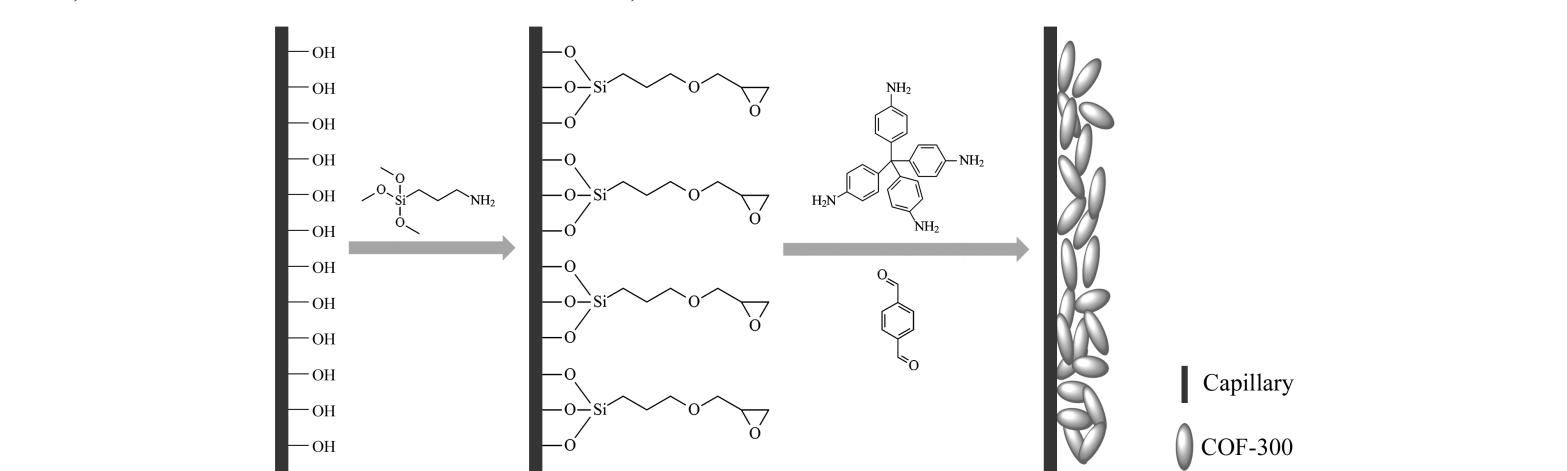
COF-300涂层毛细管柱的制备示意图^[32]^

### 1.2 整体毛细管电色谱柱的制备

整体毛细管电色谱柱因具有柱效高、柱容量大的优点在色谱分离领域得到了广泛应用^[36,37]^。相对于开管毛细管柱,COFs在整体柱领域的应用仍相对较少。与此对应,COFs整体柱的制备方法相对单一,其制备过程如下:首先在毛细管外制备COFs,之后将其与引发剂偶氮二异丁腈(AIBN)、自由基聚合单体甲基丙烯酸缩水甘油酯(GMA)及交联剂乙二醇二甲基丙烯酸酯(EDMA)引入预处理过的毛细管中,在一定条件下通过热聚合反应将COFs直接引入到有机聚合物中得到COFs整体毛细管电色谱柱。

2019年,Ji课题组^[38]^以三聚氰胺及对苯二甲醛为有机分子构建基元利用溶剂热法制备了COF SNW-1,将其与AIBN、GMA及EDMA同时引入毛细管,通过聚合反应将SNW-1嵌入整体柱,再使用纤维素酶对其进一步官能化制备了cellulase@poly(GMA-EDMA-SNW-1)整体毛细管柱。2020年,Yang课题组^[39]^以全-6-氨基-6-脱氧-*β*-环糊精和对苯二甲醛为有机分子构建基元在常温下制备了*β*-CD-COF,将其与AIBN、GMA及EDMA同时引入毛细管制备了含有*β*-CD COF的整体柱。2023年,Huang课题组^[40]^利用二-*O*-乙酰基-L-酒石酸酐修饰的Tp与对苯二胺(Pa-1)制备了手性COF CTpPa-1,然后通过AIBN、GMA及EDMA的聚合反应制备了CTpPa-1整体柱。

## 2 COFs毛细管电色谱的应用

### 2.1 环境内分泌干扰物

环境内分泌干扰物(environmental endocrine disruptors, EEDs)是一类可以通过影响天然激素的产生、分泌、分布、代谢、排泄和结合从而干扰人类及动物内分泌系统,并因此引起生殖、内分泌、免疫和神经发育发生病变的外源性化学物质^[41,42]^。目前越来越多的物质被发现具有内分泌干扰性质且广泛用于人们的日常生活,如邻苯二甲酸酯^[43]^、硝基苯酚^[44]^、双酚A^[45]^及对羟基苯甲酸酯^[46]^等。

2018年,Hu课题组^[23]^以所制备的SNW-1开管毛细管柱为分离通道对对羟基苯甲酸酯类内分泌干扰物进行了分离,结果显示,SNW-1开管毛细管柱对对羟基苯甲酸甲酯、对羟基苯甲酸乙酯、对羟基苯甲酸丙酯及对羟基苯甲酸丁酯在内的4种对羟基苯甲酸酯具有良好的分离能力。2020年,本课题组^[47]^利用以1,3,5-三(4-甲酰基苯基)苯及水合肼所制备的N_0_-COF为固定相的开管毛细管柱为分离通道对双酚A类环境内分泌干扰物进行了分离。结果表明,在最佳条件下,N_0_-COF开管毛细管柱可以实现两组共9种双酚A类化合物的基线分离。该开管毛细管柱已应用于饮料样品中双酚A及其类似物的分离测定,回收率为91.0%~112%,表明其可以应用于实际样品中双酚A及其类似物的检测。此外,本课题组^[48]^以1,3,5-均苯三甲醛及水合肼为有机分子构建基元利用相同的制备方法制备了吖嗪类ACOF-1开管毛细管柱,实现了2-硝基苯酚、4-硝基苯酚、2,4-二硝基苯酚和2,4,6-三硝基苯酚4种硝基苯酚类环境内分泌干扰物的基线分离。Chen课题组^[49]^利用1,3,5-三(4-甲酰基苯基)苯(TFPB)及BD为有机分子构建基元,采用室温原位生长法制备了TAPB-BD COF开管毛细管柱并将其应用于对羟基苯甲酸酯类物质的分离,结果表明,所制得的毛细管柱对所选目标分析物具有良好的分离性能。

### 2.2 农药

作为现代农业的一种重要生产资料,农药被广泛用于防治病虫害和提高作物产量,在保障全球粮食安全方面发挥着越来越重要的作用。然而,农药的大量使用也导致了农药残留等一系列问题,对人类、动物、水和土壤等产生了各种不可避免的负面影响^[50,51]^。因此,在食品安全和环境监测等领域,开发更为高效、灵敏和准确的农药检测技术至关重要。

Zhang课题组^[34]^将离子液体与COFs相结合建立了一种测定水果中苯并咪唑类农药的毛细管电色谱新方法。在最优实验条件下,该方法可以在19 min内实现10种苯并咪唑的分离,检出限为1.0~2.8 μg/kg,在3.5~200 μg/kg范围内具有良好的线性。对葡萄、梨及橙子等实际样品的加标回收率为85.0%~95.9%,表明该方法在水果中痕量苯并咪唑的检测方面具有良好的应用前景。Chen课题组^[33]^制备的COF-V开管毛细管柱对扑草津、扑草净胺及莠去津3种三嗪类除草剂表现出了良好的分离能力。

### 2.3 芳香族化合物

芳香族化合物(aromatic compounds)是指分子中含有苯环结构的有机化合物,广泛存在于染料、医药、农药、石化、塑料基橡胶等领域的废水中。按照所含官能团及结构特征,芳香族化合物分为多氯联苯、氯化苯酚、苯系物(苯、甲苯、乙苯和二甲苯)、多环芳烃、单芳烃和取代芳烃(硝基苯、卤代苯等)等^[52]^。由于芳香化合物已经成为土壤及水资源中普遍存在的污染物之一,因此发展新型检测技术或方法对其进行分离检测具有重要意义^[53]^。

本课题组^[22]^以制备的COF-LZU1开管毛细管柱为分离通道,以5种烷基苯、4种多环芳烃和4种苯胺为目标分析物,考察了COFs开管毛细管柱对芳香化合物的分离性能。结果表明,COF-LZU1开管毛细管柱可以分别实现3类芳香化合物的基线分离;所制备的COF-LZU1开管毛细管柱表现出了良好的稳定性,连续运行300次后分离性能没有明显的变化。Chen课题组^[31]^利用聚多巴胺的黏附性采用物理吸附法制备了多层COF-5开管毛细管柱。此开管毛细管柱被用于中性、酸性和碱性芳香族化合物的分离。

### 2.4 氨基酸

氨基酸是人体必不可少的重要组成部分,参与诸多生物过程如蛋白质、多肽和神经递质的合成等,对健康起着至关重要的作用^[54,55]^。目前,氨基酸分离分析已成为医学、制药及农业等领域不可或缺的一部分^[56,57]^。

#### 2.4.1 氨基酸非对映体

Hu课题组^[23]^于2018年通过共价结合的方式将COF SNW-1引入到毛细管内壁,并将其应用于毛细管电色谱氨基酸分离。结果显示,所制备的SNW-1开管毛细管柱对包括氨基酸在内的多种物质具有良好的分离效果,可以实现D-苯丙氨酸、D-酪氨酸及D-色氨酸3种氨基酸的基线分离。2019年,Ye课题组^[58]^利用Tp及Pa-1作为有机分子构建基元,在室温下制备的TpPa-1开管毛细管柱同样实现了D-苯丙氨酸、D-酪氨酸及D-色氨酸3种氨基酸的基线分离。2022年,本课题组^[59]^利用2,5-二羟基对苯二甲酰肼(DHzOH)及均苯三甲醛(Tf)为有机分子构建基元合成的COF Tf-DHzOH作为固定相材料,通过后修饰法制备了不同厚度的Tf-DHzOH开管毛细管柱,以此开管毛细管柱为分离通道实现了包括苯丙氨酸、酪氨酸、色氨酸、对羟基苯甘氨酸及3,4-二羟基苯丙氨酸在内的5种氨基酸的基线分离。

#### 2.4.2 氨基酸对映体

COFs毛细管电色谱在氨基酸的对映体分离领域也表现出了良好的应用前景。2020年,Wang课题组^[60]^以异氰酸酯-*β*-环糊精(isocyanate-*β*-cyclodextrin, MDI-*β*-CD)为手性选择剂,亚氨基TpPa-1 COF为基体,采用自下而上的方法合成了一种新型手性MDI-*β*-CD modified COF,进而通过原位生长法制备了手性COFs开管毛细管柱。以此开管毛细管柱作为分离通道实现了4种氨基酸的对映体分离。除了直接利用手性COFs开管毛细管柱实现对映体分离外,还可以通过在COFs开管毛细管柱中使用手性流动相的方法实现手性物质的对映体分离。2021年,Chu课题组^[61]^分别考察了以Tp及BD制备的TpBD开管毛细管柱为分离通道的毛细管电色谱法、以TpBD开管毛细管柱为分离通道结合羟丙基-*β*-环糊精(hydroxypropyl-*β*-cyclodextrin, HP-*β*-CD)为手性流动相的毛细管电色谱法及HP-*β*-CD为手性流动相的毛细管电泳法对氨基酸的分离能力。结果表明,第二种方法的分离能力及手性选择性最好,可以实现15种单氨基酸对映体的基线分离。另外,在最佳条件下,可以同时实现3种混合氨基酸外消旋体(缬氨酸、蛋氨酸、谷氨酸)的对映体分离。

### 2.5 药物

在过去的几十年里,由于药物滥用及药物不良反应等问题,人们越来越关注药物对人体健康及环境潜在的不利影响。基于此,药物分离分析已成为制药工业、生物医学、生物化学及环境保护等不同领域的重要课题^[62,63]^。目前使用的药物分离分析技术中,高效液相色谱^[64]^、气相色谱^[65]^及其与质谱联用技术应用最为广泛。除了上述技术外,毛细管电色谱技术在药物分离尤其是手性药物对映体分离方面同样做出了重要贡献^[66]^。目前,COFs毛细管电色谱在药物分离领域的应用主要分为非手性药物分离和手性药物对映体分离两个方面。

#### 2.5.1 非手性药物

Hu课题组^[23]^将所制备的COF SNW-1开管毛细管柱用于磺胺类药物、头孢菌素类药物等物质的分离,并与裸毛细管柱分离效果进行了对比。结果表明,SNW-1开管毛细管柱具有更好的分离能力。Chen课题组^[33]^所制备的COF-V开管毛细管柱实现了卡马西平和奥卡西平2种抗癫痫药的基线分离。2022年,Ye课题组^[24]^将所制备的TFA-TAPB开管毛细管柱用于氟喹诺酮类药物的分离,结果表明,TFA-TAPB开管毛细管柱可以实现7种氟喹诺酮类药物的基线分离。本课题组^[59]^制备了具有不同厚度的Tf-DHzOH开管毛细管柱,并以其为分离通道建立了分别分离9种磺胺类药物及4种四环素药物的开管毛细管柱电色谱分离新方法。结果表明,Tf-DHzOH开管毛细管柱表现出了比裸毛细管柱更好的分离效果,且Tf-DHzOH涂层厚度对分离结果具有重要影响。

#### 2.5.2 手性药物

近年来,通过使用手性COFs作为固定相,利用毛细管电色谱对手性药物对映体进行分离取得了一定的进展。Ji课题组^[38]^通过在整体柱中引入SNW-1 COF以增加外消旋体与固定相之间的相互作用,实现了包括*β*-阻滞剂、抗组胺剂及抗凝血剂在内的8对手性药物的对映体分离。Huang课题组^[40]^以氨氯地平、扑尔敏、联糠醛及布洛芬为目标分析物,考察了所制备的手性COF CTpPa-1开管毛细管柱的对映体分离能力。结果显示,所制备的CTpPa-1开管毛细管柱对上述4种手性药物的对映体均可实现基线分离,且表现出了良好的重复性和稳定性。Wang课题组^[60]^利用环糊精对有机分子构建基元进行修饰的方法将环糊精引入到COFs孔径中,以此COFs制备的开管毛细管柱实现了阿替洛尔、拉贝洛尔、索他洛尔及塞利洛尔4种*β*-阻滞剂的对映体分离。Ji课题组^[67]^利用框架结构中含有*β*-环糊精的*β*-CD COF作为手性固定相分别实现了6种手性药物对映体的基线分离,且具有良好的重现性。在此之后,该课题组^[21]^在*β*-CD COF的基础上,通过后修饰制备了2种COFs开管毛细管柱,并选取手性药物作为目标物对其对映体分离能力进行了比较。结果发现引入官能团可以改变*β*-CD COF的性质,并调节其手性识别能力,可以更好地实现对映体的分离。

发展新型手性COFs及COFs开管毛细管柱制备方法,对满足对映体分离多样性的需求及促进COFs在毛细管电色谱手性分离领域的应用具有重要作用。基于此,本课题组^[30]^通过自下而上的策略,设计并合成了一种具有高结晶度和良好化学及热稳定性的新型手性DA-TD COF,并采用手性DA-TD COF作为开管毛细管电色谱的手性固定相进行手性药物对映体分离。结果显示,手性DA-TD COF开管毛细管柱对8种手性药物的对映体具有良好分离性能,且连续运行200次后分离效率无明显变化。为了优化COFs开管毛细管柱的制备过程,本课题组^[68]^利用(*R*)-2-(3-氯-2-羟丙基)-1,3,5-三(4-氨基苯基)苯(CB)及DA,通过一步原位生长法在室温下制备了手性CB-DA-COF开管毛细管柱并将其作为分离通道用于手性药物的对映体分离。该方法既不需要有机分子构建基元对毛细管进行预修饰,也不需要苛刻的反应条件,显著缩短了手性COFs涂层的制备时间。结果表明,该手性开管毛细管柱对特布他林、普萘洛尔、苯肾上腺素、维拉帕米、去甲肾上腺素和异丙肾上腺素等6种手性药物的对映体具有良好的分离能力。

## 3 分离机理

虽然COFs毛细管电色谱仍处于起步阶段,但其已经在众多领域表现出了良好的分离能力,具有巨大的应用前景。为了促进COFs在毛细管电色谱领域的应用及更好地设计制备具有高效分离能力的COFs及基于COFs固定相的毛细管电色谱柱,科研工作者通过实验设计及计算模拟等手段对基于COFs固定相的毛细管电色谱分离机理进行了研究^[23,27,68]^。目前,普遍认为基于COFs固定相的毛细管电色谱的良好分离能力主要得益于以下两个方面:(1)COFs孔的尺寸排阻作用。由于有机分子构建基元和侧链的尺寸不同,形成的COFs将具有不同的孔径及拓扑结构,在作为固定相进行分离时只允许小于其孔径的目标分析物通过。与之对应,尺寸较大的目标分析物则难以通过COFs孔,只能从COFs固定相材料表面或空隙中通过^[30]^。(2)目标分析物与COFs框架或侧链之间的相互作用,如疏水相互作用、氢键相互作用、*π-π*相互作用等。由于COFs往往含有烷烃侧链、芳香结构、氧和/或氮等电负性较大的原子,因此可能与目标分析物之间发生多种相互作用^[20,68]^。

2022年,本课题组^[68]^分别制备了高结晶度的CB-DA COF开管毛细管柱及无定型CB-DA-COF开管毛细管柱,并对其手性分离效果进行了对比。结果表明,高结晶度的CB-DA COF固定相对手性药物具有良好的分离能力,而无孔结构的无定型CB-DA-COF固定相并没有手性分离能力,证明COFs的孔结构对分离效果具有重要贡献。利用AutoDock软件通过遗传算法对手性CB-DA COF与目标分析物之间的结合能进行了计算,结果表明,对于对映体分离效果较好的药物,其两种对映体与手性CB-DA COF孔内的结合能差距较大。Ji课题组^[21]^利用AutoDock软件通过分别计算手性COFs与两种对映体之间的结合能大小(Δ*G*)及两者差值(ΔΔ*G*),研究了改性后的*β*-CD COF对二氢吡啶和氟喹诺酮类药物的手性识别机制和分子间相互作用。计算结果表明,在手性COFs引入的官能团和对映体之间有更多的相互作用,从而提高了*β*-CD COFs的手性识别能力。2023年,Huang课题组^[40]^利用分子对接技术研究了CTpPa-1整体毛细管柱在进行手性分离时CTpPa-1与手性化合物之间的相互作用。结果表明,对映体与手性COF CTpPa-1之间的结合自由能差值与对映体选择性因素有关,丰富的乙酰氧基、腈基及苯环是手性COF CTpPa-1整体毛细管柱对映体分离能力的来源。

## 4 总结与展望

综上所述,COFs已在毛细管电色谱中得到了一定的应用并展现出了良好的应用前景。但是,COFs在毛细管电色谱领域的应用尚处于起始阶段,仍存在许多亟待深入研究和解决的问题。首先,大多数COFs的合成条件较为苛刻(高温、密封、反应时间冗长等),不适用于原位合成,只能通过后修饰的方式制备COFs毛细管柱,限制了它们在毛细管电色谱中的应用。此外,基于COFs固定相的毛细管电色谱分离机理研究仍不够深入,难以确定分子尺寸选择作用及COFs与目标分析物之间的相互作用的贡献大小,无法通过COFs的结构及孔径尺寸来判断其对目标分析物的分离能力,限制了开发分离效率高、分离能力强的毛细管电色谱方法。鉴于此,我们认为今后应围绕以下几方面开展基于COFs固定相的毛细管电色谱研究:(1)针对在复杂体系中分离测定目标组分的要求,利用化学信息学方法设计构造有可能用于毛细管电色谱的COFs,减少合成COFs的盲目性,提高制备COFs毛细管电色谱柱的效率;(2)开发条件更为温和的COFs制备方法,以满足简便快速制备性能更加优异的COFs毛细管柱的要求;(3)深入开展基于COFs固定相的毛细管电色谱分离机理的研究,为建立适用于复杂样品分离分析的毛细管电色谱方法提供理论指导。
